# Resolution therapy: Harnessing efferocytic macrophages to trigger the resolution of inflammation

**DOI:** 10.3389/fimmu.2022.1021413

**Published:** 2022-10-28

**Authors:** Philippe Saas, Mathieu Vetter, Melissa Maraux, Francis Bonnefoy, Sylvain Perruche

**Affiliations:** ^1^ University Bourgogne Franche-Comté, INSERM, EFS BFC, UMR1098, RIGHT, Interactions Hôte-Greffon-Tumeur/Ingénierie Cellulaire et Génique, LabEx LipSTIC, Besançon, France; ^2^ MED’INN’Pharma, Besançon, France

**Keywords:** macrophage, apoptotic cells, efferocytosis, resolution of inflammation, inflammation, TGF-β, non-resolving inflammation, macrophage reprogramming

## Abstract

Several chronic inflammatory diseases are associated with non-resolving inflammation. Conventional anti-inflammatory drugs fail to completely cure these diseases. Resolution pharmacology is a new therapeutic approach based on the use of pro-resolving mediators that accelerate the resolution phase of inflammation by targeting the productive phase of inflammation. Indeed, pro-resolving mediators prevent leukocyte recruitment and induce apoptosis of accumulated leukocytes. This approach is now called resolution therapy with the introduction of complex biological drugs and cell-based therapies. The main objective of resolution therapy is to specifically reduce the duration of the resolution phase to accelerate the return to homeostasis. Under physiological conditions, macrophages play a critical role in the resolution of inflammation. Indeed, after the removal of apoptotic cells (a process called efferocytosis), macrophages display anti-inflammatory reprogramming and subsequently secrete multiple pro-resolving factors. These factors can be used as resolution therapy. Here, we review the different mechanisms leading to anti-inflammatory reprogramming of macrophages after efferocytosis and the pro-resolving factors released by these efferocytic macrophages. We classify these mechanisms in three different categories: macrophage reprogramming induced by apoptotic cell-derived factors, by molecules expressed by apoptotic cells (*i.e*., “eat-me” signals), and induced by the digestion of apoptotic cell-derived materials. We also evoke that macrophage reprogramming may result from cooperative mechanisms, for instance, implicating the apoptotic cell-induced microenvironment (including cellular metabolites, specific cytokines or immune cells). Then, we describe a new drug candidate belonging to this resolution therapy. This candidate, called SuperMApo, corresponds to the secretome of efferocytic macrophages. We discuss its production, the pro-resolving factors present in this drug, as well as the results obtained in experimental models of chronic (e.g., arthritis, colitis) and acute (e.g., peritonitis or xenogeneic graft-*versus*-host disease) inflammatory diseases.

## 1 Introduction

Inflammation is a natural protective response to fight against any aggression, such as infections. Under physiological conditions, the resolution phase of inflammation allows the body to stop inflammation, and promotes tissue repair to return to homeostasis. Carl Nathan and Aihao Ding were the first to propose the concept of non-resolving inflammation (1). Thus, alterations in the resolution phase of inflammation lead to uncontrolled chronic inflammation responsible for tissue damage. This non-resolving inflammation is encountered in several chronic inflammatory diseases, including atherosclerosis, asthma, inflammatory bowel diseases (IBD), multiple sclerosis (MS), rheumatoid arthritis (RA), as well as cancer (1). Although these chronic inflammatory disorders result from various pathogenic mechanisms, they share this non-resolving inflammation (1). Nevertheless, these chronic diseases are not always controlled by current treatments and development of new therapeutic approaches is urgently required (as recently discussed in an editorial on inflammatory rheumatic diseases (2)).

Resolution pharmacology is a new therapeutic approach based on the use of resolution mediators (3–5). The idea is to stimulate the resolution phase in order to accelerate (or achieve) the return to homeostasis. Indeed, a delay in the resolution can extend the duration of the pro-inflammatory response resulting in tissue damage, which in turn, prolongs the inflammatory state. Among the resolution mediators used in resolution pharmacology, one may evoke specialized pro-resolving lipid mediators (SPM, including lipoxins, resolvins, protectins, and maresins) (6, 7), or proteins (e.g., Annexin-A1 (8), DEL-1 (9) (for reviews please see (5, 10, 11)). Recently, the list of the candidates has been extended to complex biological drugs (e.g., secretomes released by particular cells) or cell-based therapies. This is why the terms “resolution therapy” or “resolution therapeutics” (12) are preferable to describe this therapeutic arsenal able to stimulate the resolution of inflammation.

A critical step in the switch from the initiation/onset phase to the resolution one is mediated by macrophages performing the elimination of apoptotic neutrophils (13–15). This process is called efferocytosis. Efferocytosis enables macrophages to shift from a pro-inflammatory to a pro-resolving function (16–18). This shift, named macrophage reprogramming, consists of the decreased synthesis of pro-inflammatory factors (e.g. IL-1β, IL-12 or TNF) (19, 20) associated with the concomitant increased production of anti-inflammatory mediators (e.g. TGF-β, prostaglandin-E2 [PGE-2] (19). Most of the resolution mediators are produced by pro-resolving macrophages (17, 18). In the current review, we want to focus on these pro-resolving factors released by macrophages after efferocytosis. Before that, we propose to evoke the mechanisms triggered by efferocytosis that stimulate macrophage reprogramming through a pro-resolving profile. Recently, others (17, 18) and we (21–24) have reviewed the different mechanisms leading to anti-inflammatory macrophage reprogramming after efferocytosis. Here, we will focus on the most recent findings. Then, after this part on macrophage reprogramming and pro-resolving factors generated by these cells after efferocytosis, we will describe and discuss a new drug candidate for resolution therapy developed by our laboratory.

## 2 Efferocytosis as a critical step triggering the resolution phase of inflammation and the release of pro-resolving factors

Macrophages represent heterogeneous cells with different phenotypes. This heterogeneity is encountered even in a given tissue, at steady state, but also during pathogenic situations. The origin of macrophage may influence this heterogeneity (25). Indeed, macrophages may arise from hematopoietic progenitors during embryogenesis and then become tissue-resident macrophages. These tissue-resident macrophages exhibit a self-renew capacity. Macrophage proliferation is critical for maintaining a tissue-resident macrophage pool and participating in tissue homeostasis or protection (26–28). These tissue-resident macrophages are regularly exposed to apoptotic cells generated during normal cell turnover and this exposure imprints an anti-inflammatory program (29, 30). Macrophages may also differentiate from blood monocytes during inflammation (16, 31). Macrophages may therefore exert both pro- or anti-inflammatory functions. Pro-inflammatory macrophages play a critical role in the onset phase of inflammation, while anti-inflammatory macrophages are involved in the resolution phase of inflammation. Furthermore, macrophage heterogeneity may also depend on their tissue location (16).

In addition to their heterogeneity, another salient property of these cells is their extreme plasticity (16, 32). Macrophages may exert a huge “spectrum” of functions characterized by an array of different phenotypes (33). The two extreme polarized phenotypes of this continuum were initially called M1 and M2 (34). These terms tend to be abandoned nowadays (35), in particular after the description of this “spectrum” model (33). Nevertheless, this M1/M2 classification (34) can be used when describing macrophages in culture during a well-defined condition of stimulation (e.g., the presence of IL-4). The M1 phenotype characterizes pro-inflammatory macrophages involved in anti-infectious responses, and during the onset phase of inflammation. Cells of this subset are also sometimes called “classically” activated macrophages. In contrast, the M2 phenotype represents anti-inflammatory (“alternatively” activated) macrophages. This phenotype can be subdivided into several subtypes with diverse functions. This includes, for instance, immunosuppressive tumor-associated macrophages (TAM), or the pro-resolving macrophages participating in the resolution phase of inflammation. However, the transcriptomic signature of mouse pro-resolving macrophages differs from those of “M2-like” macrophages (36). This attests that pro-resolving macrophages of the resolution phase belong to a specific macrophage subtype, consistent with the “spectrum” model.

**Figure 1:**
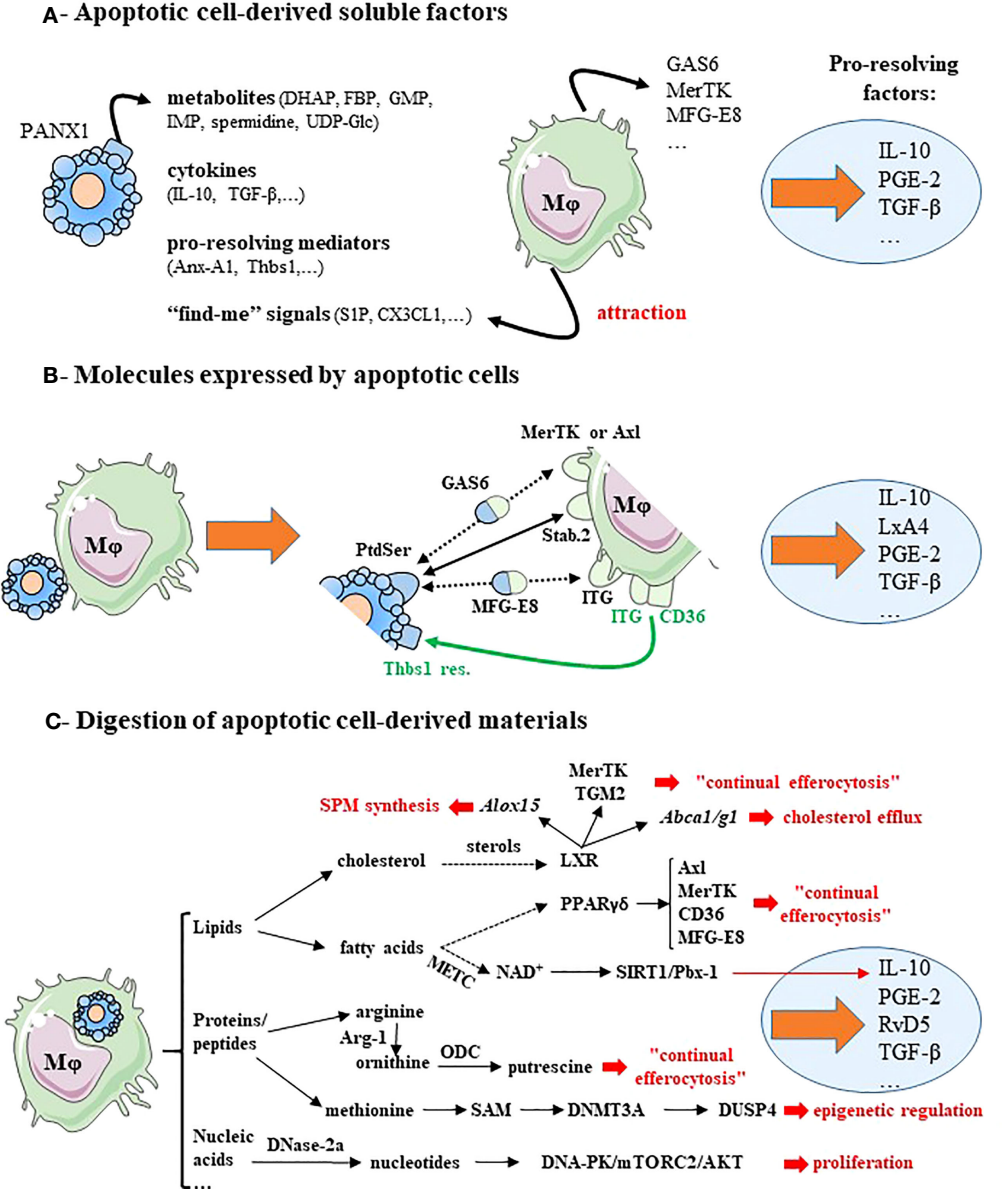
Macrophage reprogramming after efferocytosis may result from apoptotic cell-derived factors, surface molecules expressed by apoptotic cells or metabolites generated by apoptotic cell digestion. **(A)** Soluble factors released by apoptotic cells induce pro-resolving macrophage reprogramming and lead to pro-resolving factor secretion. Apoptotic cells release four kind of factors: cellular metabolites *via* the Pannexin-1 channels (PANX1), anti-inflammatory cytokines, pro-resolving mediators and “find-me” signals. These “find-me” signals affect tissue-resident macrophages locally, and diffuse to target monocyte-derived macrophages and attract them. These mediators stimulate the expression of efferocytic receptors (MerTK) and opsonins (e.g., GAS6 or MFG-E8). **(B)** Molecules expressed by cells becoming apoptotic promote macrophage reprogramming and induce the synthesis of pro-resolving factors. This is illustrated by the expression of the chief “eat-me” signal phosphatidylserine (PtdSer) that interacts directly with efferocytic receptors (e.g., Stabilin-2 [Stab.2]), or indirectly *via* bi-functional opsonins (e.g., GAS6 or MFG-E8) and with the efferocytic receptors MerTK or integrin receptors (ITG)(black lines). CD36 in association with integrin receptors (ITG, ITGB3 or ITGA5) recognizes thrombospondin-1 residues (Thbs1 res.) at the apoptotic cell surface (green lines). **(C)** Cellular metabolites resulting from apoptotic cell-derived materials stimulate macrophage reprogramming and the secretion of pro-resolving factors. In addition, these metabolites trigger several signaling pathways, implicated in efficient reprogramming including: macrophage proliferation, epigenetic regulation, continual efferocytosis, cholesterol efflux and specialized pro-resolving lipid mediator (SPM) synthesis (text in red font). Dotted arrows correspond to a suspected and indirect link. Pro-resolving factors are identified by light blue circles. Gene names are written in italics. For more details, please see the text. Abbreviations: ABC, ATP-binding cassette transporters; Alox15, arachidonate 15-lipoxygenase; Anx-A1, annexin-A1; Arg-1, arginase-1; CX3CL1, fractalkine; DHAP, dihydroxyacetone phosphate; DNA-PK, DNA-dependent protein kinase; DNase-2a, deoxyribonuclease-2a; DNMT3A, DNA methyltransferase-3A; DUSP4, dual-specific phosphatase 4; FBP, fructose-1,6-biphosphate; GAS6, growth arrest-specific protein 6; GMP, guanosine-5′-monophosphate; IMP, inosine-5′-monophosphate; ITG, integrin receptors;, LxA4, lipoxin-A4; LXR, liver X receptor; METC, mitochondrial electron transport chain; Mφ, macrophage; MFG-E8, milk fat globule-EGF factor 8; NAD^+^, nicotinamide adenine dinucleotide coenzyme; mTORC2, mammalian Target Of Rapamycin complex-2; ODC, ornithine decarboxylase; PANX1, pannexin-1; PGE-2, prostaglandin-E2; PPAR, peroxisome proliferator-activated receptor; PtdSer, phosphatidylserine; RvD5, resolvin D5; S1P, sphingosine-1-phosphate; SAM, S-adenosylmethionine; SIRT1, sirtuin-1; Stab.2, stabilin-2; TGF-β, transforming growth factor-β; TGM2, transglutaminase-2; Thbs1, thrombospondin-1; Thbs1 res., thrombospondin-1 residues; UDG-Glc, uridine-diphosphate-glucose. This figure was depicted, in part, by using Servier Medical Art, https://smart.servier.com/.

As mentioned above, macrophages are plastic cells highly sensitive to their microenvironment. Cells dying by apoptosis create an anti-inflammatory microenvironment that may affect neighboring macrophages. Thus, soluble factors released by apoptotic cells may stimulate macrophage reprogramming (**Figure 1A**). These factors released by apoptotic cells have been proposed to be used therapeutically to promote the resolution of inflammation (8, 37, 38). The administration of apoptotic cells themselves has been tested in experimental models of chronic inflammatory diseases (e.g., collagen-induced arthritis [CIA] (39), as well as in clinical settings (40, 41). We were the first in 2001 to propose the use of apoptotic cells as a cell-based therapy approach (42). However, this is out of the scope of this review (for recent reviews please refer to (21, 24, 43). Here, we want to focus on the contribution of macrophages to the resolution therapy.

**Figure 2:**
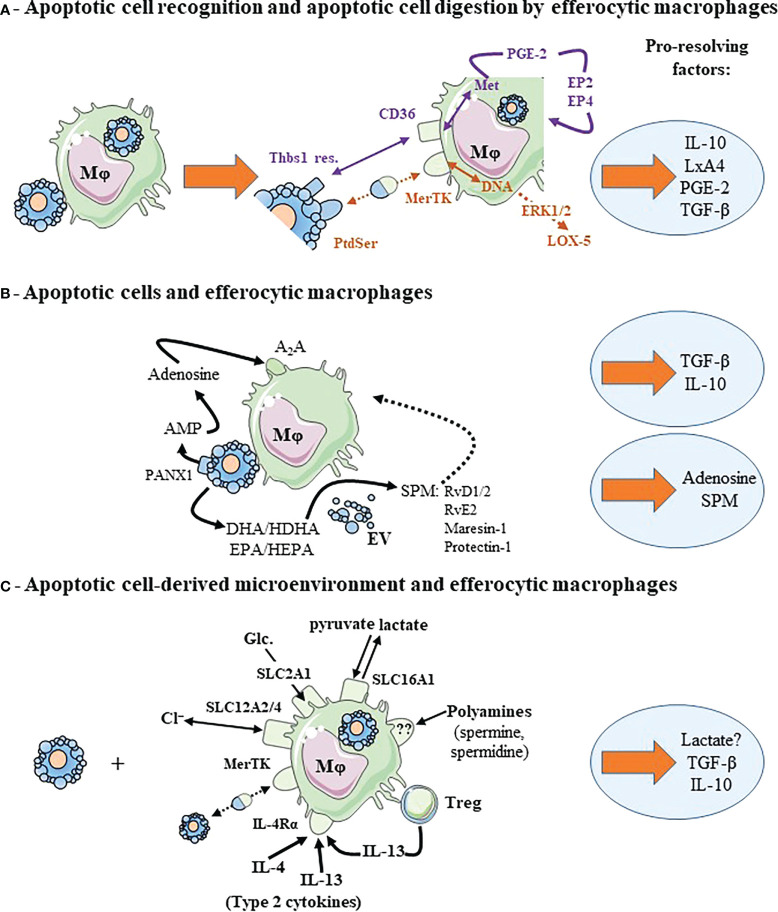
Cooperative mechanisms implicating efferocytic macrophages contributes to macrophage reprogramming and to the release of pro-resolving factors. **(A)** Recognition of apoptotic cell surface molecules by their cognate receptors and metabolites generated from digested apoptotic cells cooperate to induce macrophage reprogramming and pro-resolving factor release. Two examples are given corresponding to two different colors: DNA and recognition of PtdSer by MerTK in brown color, and methionine (Met) and recognition of thrombospondin-1 residues (Thbs1 res.) by CD36 in purple. We illustrated also the autocrine loop leading to TGF-β secretion implicating PGE-2 and its receptors, EP2 and EP4. **(B)** Efferocytic macrophages cooperates with apoptotic cells. Each cell contributes to macrophage reprogramming and the secretion of pro-resolving factors. This is the case of adenosine conversion by macrophages from AMP (adenosine mono-phosphate) released in the extracellular milieu by apoptotic cells through the Pannexin-1 (PANX1) channels. Apoptotic neutrophils or extracellular vesicles (EV) issued from these apoptotic neutrophils participate in the transcellular biosynthesis of specialized pro-resolving lipid mediators (SPM) by macrophages. Apoptotic neutrophils or EV provide SPM precursors (not necessarily DHA or EPA, but other intermediate precursors [HDHA and HEPA, respectively]) that are transformed in SPM by efferocytic macrophages. **(C)** Efferocytic macrophages may require signals from their microenvironment to acquire a pro-resolving profile and secrete pro-resolving factors. This microenvironment may correspond to ions (chloride, Cl^−^), Glucose (Glc.) or their metabolites regulated through solute carrier transporter (SLC) exchange, polyamines, the presence of type 2 cytokines or regulatory T cells (Treg). Dotted arrows correspond to a suspected and indirect link. Pro-resolving factors are identified by light blue circles. For more details, please see the text. Other abbreviations: A2A, Adenosine 2A receptor; DHA, docosahexaenoic acid; EPA, eicosapentaenoic acid; EP, prostaglandin-E2 receptor; ERK, extracellular signal-regulated kinase 1/2; HDHA, hydroxy-docosahexaenoic acid; HEPA, hydroxy-eicosapentaenoic acid; IL-4Rα, interleukin-4 receptor-alpha; LxA4, Lipoxin A4; Mφ, macrophage; PGE-2, prostaglandin-E2; RvD1/2, resolvin D1 and resolvin D2; RvE2, resolvin E2. This figure was depicted, in part, by using Servier Medical Art, https://smart.servier.com/.

Mechanisms leading to the control of macrophage reprogramming and those triggering the secretion of pro-resolving factors after efferocytosis are now beginning to be elucidated. Two main types of mechanisms have been identified so far. First, mechanisms delivered by apoptotic cells themselves (**Figures 1A, B**). This corresponds to soluble factors released by these dying cells (**Figure 1A**) and the direct interactions implicating cognate receptors expressed by apoptotic cells/bodies (including apoptotic cell-derived extracellular vesicles) and macrophages, respectively (**Figure 1B**). Second, the digestion of apoptotic cell-derived materials by efferocytic macrophages stimulates their reprogramming, and then the release of pro-resolving factors (**Figure 1C**). A synergy between these two mechanisms also exists (**Figure 2A**). A cooperation between apoptotic cells and macrophages before the engulfment of apoptotic cells may also generate pro-resolving factors (**Figure 2B**), such as adenosine. This nucleoside derives from adenosine monophosphate (AMP) released by apoptotic cells, which is then converted to adenosine by efferocytic macrophages (44). Production of SPM may also involve the cooperation of apoptotic cells and macrophages (6, 45). A last possibility to allow macrophages to generate pro-resolving mediators may result from the microenvironment in which the cells die (**Figure 2C**). Before discussing these mechanisms participating in macrophage reprogramming, we have to mention the three pioneer studies that initiated the elucidation of macrophage reprogramming and its role in the resolution of inflammation. The first study has proposed the critical role of apoptotic neutrophil elimination by macrophages to stop inflammation (13). The other two reported the synthesis by efferocytic myeloid cells of IL-10 (46) and TGF-β (19), two major anti-inflammatory cytokines associated with macrophage reprogramming. These cytokines are among the main factors studied that contribute to macrophage reprogramming.

### 2.1 Macrophage reprogramming induced by soluble factors released by apoptotic cells

Apoptotic cells create a local transient immunosuppressive microenvironment to prevent undesirable immune responses. This microenvironment does not affect only macrophages. Apoptotic cells may prevent the attraction of different immune cells by neutralizing inflammatory chemokines *via* the upregulation of CCR5 expression (47). In addition, apoptotic cells release soluble factors, including anti-inflammatory cytokines (e.g., TGF-β (48) or IL-10 (49), pro-resolving mediators (e.g., annexin-A1 (38) or thrombospondin-1 [Thbs1] (50)), as well as cellular metabolites (e.g., spermidine (37)). Some of these factors may induce macrophage reprogramming. For instance, together with M-CSF, IL-10 participates in the differentiation of human anti-inflammatory macrophages (named M2c according to the old nomenclature) (51). IL-10 upregulates the MerTK efferocytic receptor and the release of the bridging molecule, GAS6 (51), thereby promoting a higher capacity to clear apoptotic cells (52) (*i.e*., “continual efferocytosis”, see below). Apoptotic cells have been also reported to secrete “find-me” signals in order to attract professional phagocytes (e.g., monocyte-derived macrophages) to the site where the cells die. Among these “find-me” signals, some of them have immunomodulatory properties that may stimulate macrophage reprogramming (53). For instance, the lipid sphingosine-1-phosphate induces M2 macrophage reprogramming with an increased production of IL-10 and PGE-2 (54). The “find-me” signal, fractalkine (also known as CX3CL1), enhances macrophage efferocytosis *via* the increased secretion of milk fat globule-EGF factor 8 (MFG-E8) (55), and also stimulates TGF-β production by macrophages (53). Thus, the microenvironment created by apoptotic cells may induce macrophage reprogramming and generate pro-resolving factors (**Figure 1A**).

### 2.2 Macrophage reprogramming induced by molecules expressed by apoptotic cells

After the emission of the “find-me” signals, apoptotic cells express “eat-me” signals that are recognized by receptors present on efferocytic cells. This promotes apoptotic cell engulfment (56). The interactions between “eat-me” signals expressed by apoptotic cells and their cognate receptors expressed by macrophages can stimulate macrophage reprogramming. Here, we will focus on some efferocytic receptors that deliver a reprogramming signal in macrophages. We will not be exhaustive in the description of the numerous interactions occurring between apoptotic cells and macrophages. For that, we recommend the following recent reviews (17, 56–58).

We will take the example of the main “eat-me” signal, phosphatidylserine (PtdSer) that is normally confined to the inner leaflet of the plasma membrane of viable cells. Apoptotic cells externalize PtdSer to the outer leaflet, and thus, express high levels of PtdSer at their cell surface. The pioneer works of Fadok and Henson identified the recognition of PtdSer by macrophages as critical for apoptotic cell removal (59), the production of TGF-β (19), and the resolution of inflammation (60). Masking this PtdSer present at the cell surface of apoptotic cells prevents both efferocytosis and its associated anti-inflammatory response (61). Several receptors recognizing PtdSer have been identified [for review (53, 56–58)]. Some interactions between “eat-me” signals and their cognate receptors require bi-functional soluble bridging molecules (named also opsonins). The Complement component C1q is an opsonin binding to different “eat-me” signals expressed by apoptotic cells, including PtdSer (56). This opsonin is recognized by several macrophage receptors, such as LRP1 (CD91), CR1 (CD35) or SCARF-1 (56, 62). The recognition of apoptotic cells *via* C1q induces anti-inflammatory macrophage reprogramming with the production of IL-10 (63). Axl and MerTK, two members of the TAM receptor family, recognize indirectly PtdSer *via* the opsonins, GAS6 or protein S (58). The stimulation of MerTK by apoptotic cells induces the translocation of lipoxygenase-5 (LOX-5) from the nucleus to the cytosol leading to the synthesis of the SPM, lipoxin-A4 (LxA4) derived from arachidonic acid (AA) (64). This connects apoptotic cell “eat-me” signal, macrophages and resolution mediators SPM. Furthermore, LxA4 increases TGF-β secretion by mouse macrophages (65), connecting SPM to anti-inflammatory cytokines. Another bridging molecule, called MFG-E8, allows the interactions between PtdSer and integrin receptors (*i.e*., α_v_β_3_ [ITGB3], also known as the vitronectin receptor (66) or α_v_β_5_ [ITGA5]) expressed by macrophages (53). These integrin receptors coupled with CD36 interact with the Thbs1 residues expressed at the cell surface of apoptotic cells. The engagement of these integrin receptors by apoptotic cells induces the production of TGF-β by macrophages (53). Stabilin-2, another PtdSer receptor involved in efferocytosis, induces TGF-β secretion after apoptotic cell recognition (67). Moreover, CD300 family members representing other PtdSer receptors may stimulate IL-10 production by macrophages (53). Overall, surface molecules expressed by apoptotic cells −in association or not with opsonins− favor macrophage reprogramming with the secretion of anti-inflammatory cytokines. This step is critical as attested by systemic autoimmune diseases occurring in MerTK-deficient mice (68), or when PtdSer is masked (61).

### 2.3 Macrophage reprogramming induced by the digestion of engulfed apoptotic cells

The third step involved in the elimination of apoptotic cells −after the emission of “find-me” signals and the expression of “eat-me” signals− is the digestion or degradation of apoptotic cell derived-materials. The digestion of these apoptotic cell-derived materials (e.g., lipids, proteins/peptides, or nucleic acids) by macrophages after efferocytosis leads to a huge number of cellular components that should be transformed (*i.e*., metabolized) and recycled by macrophages, or alternatively excreted to avoid their accumulation. This process of apoptotic cell digestion by macrophages is critical to prevent exacerbated autoimmune responses (69, 70) and to trigger the resolution of inflammation. This step is necessary for macrophages to acquire a pro-resolving and pro-repair profile (53, 71). Apoptotic cell-derived components generated by apoptotic cell degradation in phagolysosomes of macrophages may be implicated in macrophage reprogramming.

#### 2.3.1 Lipid metabolism

The digestion of apoptotic cell-derived lipids by macrophages may lead to the accumulation of cellular components, including cholesterol and fatty acids. These apoptotic cell-derived lipids may affect macrophage functions (70–72), including both macrophage reprogramming and the capacity to continue to eliminate apoptotic cells over time. This process has been called by Ira Tabas “continual efferocytosis” (17, 73). Cholesterol represents one of the major apoptotic cell-derived component. The lysosomal acid lipase is critical to hydrolyze cholesterol in the lysosomes after efferocytosis (70). This allows macrophages to produce anti-inflammatory (oxy)sterols that are required for optimal LXR activation. This LXR pathway stimulates then cholesterol efflux *via* the synthesis of ATP-binding cassette (ABC) transporters (70). Macrophages, like most of the cells of our body, lack the capacity to breakdown cholesterol. This efflux of cholesterol *via* ABC transporters (*i.e*., ABCA1 and ABCG1) is thus critical for macrophage homeostasis. Apoptotic cells induce their own clearance *via* the LXR pathway that increases the expression of efferocytic receptor MerTK (72). Sterols derived from apoptotic cell-derived cholesterol may activate LXR in human efferocytic macrophages (74). In turn, LXR activation induces the upregulation of arachidonate 15-lipoxygenase (ALOX15). This enzyme participates in the resolution of inflammation by triggering the synthesis of resolvin D5 (RvD5) (74). The stimulation of the LXR/retinoic acid receptor-α (RAR-α) pathway enhances also the uptake of apoptotic cells through the efferocytic receptor, transglutaminase-2 (TGM2) (75). Cholesterol derivatives issued from digested apoptotic cells may also promote this TGM2 pathway, which may participate in “continual efferocytosis”. Until now, cholesterol and its derivatives that accumulate after efferocytosis have not been metabolically traced as coming specifically from apoptotic cells.

An enrichment of long-chain fatty acids is also found in efferocytic macrophages using unbiased liquid chromatography-tandem mass spectrometry (71). Fatty acid breakdown from ingested apoptotic cells may be responsible for this enrichment. These fatty acids may stimulate mitochondrial respiration and may be involved in a non-canonical anti-inflammatory signaling pathway. This pathway requires an intact mitochondrial electron transport chain and involves the nicotinamide adenine dinucleotide NAD^+^ coenzyme, the Sirtuin-1 signaling protein, and the transcription factor Pbx-1 that controls *Il-10* gene expression (71). Fatty acids generated from ingested apoptotic cells could be also potential activators of peroxisome proliferator-activated receptors (PPAR) (76). PPARγ and PPARδ have been shown to regulate macrophage reprogramming after efferocytosis (77–80). These nuclear receptors induce an increased expression of efferocytic receptors (e.g., Axl, MerTK or CD36), and the release of opsonins (e.g., MFG-E8) facilitating the uptake of apoptotic cells (77, 79–81). In addition, LXR and PPAR have been shown to antagonize the prototypical pro-inflammatory transcription factor, NF-κB (82, 83), which regulates the synthesis of pro-inflammatory cytokines, IL-6 or TNF. This participates in macrophage reprogramming by blocking pro-inflammatory cytokine production. While the LXR pathway is clearly anti-inflammatory in mouse macrophages, this pathway may be also pro-inflammatory in human macrophages (23). Nevertheless, lipid metabolism resulting from apoptotic cell degradation may participate in macrophage reprogramming after efferocytosis and the production of pro-resolving factors (**Figure 1C**).

#### 2.3.2 Amino acid metabolism

Amino-acid levels increase after efferocytosis as a consequence of apoptotic cell-derived protein/peptide degradation. This concerns, in particular arginine, ornithine, lysine and methionine, while no increase of alanine and glycine is detected (73, 84). Ornithine may result from apoptotic cell-derived arginine metabolized by arginase-1 (73). An increased arginase-1 activity in mouse efferocytic macrophages has been confirmed in another study (84). Then, ornithine may be transformed *via* ornithine decarboxylase (ODC) into putrescine. An increase of ODC activity after efferocytosis is detected (73). The arginase-1/ODC/putrescine pathway is implicated in “continual efferocytosis” in mouse macrophages (73). In contrast, the next metabolites after putrescine in the polyamine pathway (*i.e*., spermidine and spermine, respectively) are not required for “continual efferocytosis” (73). The situation is a little bit more complex for human macrophages. Indeed, in contrast to data obtained in mouse pro-resolving macrophages, arginase-1 is not a marker of human pro-resolving macrophages (85). However, apoptotic-cell derived ornithine and its metabolite putrescine both contribute also to “continual efferocytosis” in human macrophages (73).

Another amino acid, methionine, is generated by the phagosomal degradation of apoptotic cell-derived proteins or peptides. Methionine is then converted into S-adenosylmethionine that is used by a DNA methyltransferase (DNMT) called DNMT3A. This enzyme transfers methyl groups to regulatory DNA regions leading to the suppression of gene transcription. Thus, dual-specific phosphatase 4 is repressed by DNMT3A after efferocytosis. This pathway is involved in macrophage reprogramming after efferocytosis by inducing the production of PGE2 and TGF-β (12). This work identifies an epigenetic regulation mechanism by which apoptotic cell-derived materials give macrophages a pro-resolving phenotype. This epigenetic regulation may induce prolonged anti-inflammatory macrophage reprogramming.

#### 2.3.3 Nucleic acid degradation

Digestion of apoptotic cell-derived nucleic acids is critical to avoid exacerbated autoimmune responses (69). The levels of nucleotides resulting from nucleic acid degradation increase in efferocytic macrophages (84). This is the case of cytosine for instance (84). Apoptotic cell-derived nucleotides after the degradation of DNA by cellular deoxyribonuclease-2a activate the DNA-dependent protein kinase/mTORC2/phospho-AKT proliferation pathway (86). Thus, macrophages proliferate in response to efferocytosis. Efferocytosis-induced proliferation is required for macrophages to acquire their pro-resolving functions. Macrophages undergoing efferocytosis-induced proliferation continue to eliminate apoptotic cells and produce the two key anti-inflammatory cytokines associated with efferocytosis, TGF-β and IL-10 (86).

Overall, controlled degradation of apoptotic cell materials is required to drive the acquisition of pro-resolving properties and to prevent chronic inflammation. Metabolites generated from ingested apoptotic cells may trigger signaling pathways leading to pro-resolving factors (**Figure 1C**).

### 2.4 Macrophage reprogramming induced by cooperative mechanisms

In the next three paragraphs, we will discuss the cooperation of different mechanisms leading to the production of pro-resolving factors. The first associates mechanisms already described (2.3), while the other two cooperative mechanisms require a partnership between cells (*i.e*., apoptotic cells and macrophages), or with their microenvironment (e.g., cellular metabolites, specific cytokines or immune cells). Understanding these cooperative mechanisms is highly pertinent for the discussion of the new pro-resolving drug candidate SuperMApo (section 3).

Macrophage reprogramming can be stimulated by the synergistic action of apoptotic cell recognition by macrophage efferocytic receptors and the degradation of apoptotic cell-derived components (**Figure 2A**). Indeed, the combined action of apoptotic cell recognition by macrophage receptor CD36 and the generation of methionine by phagolysosomal degradation of apoptotic cells are necessary for PGE2 and TGF-β production (12). TGF-β production results from an autocrine loop implicating PGE2 and its two receptors prostaglandin-E2 receptors 2 and 4 (EP2 and EP4) present on macrophage surface (12). In the same way, the simultaneous recognition of apoptotic cells by MerTK expressed by macrophages and the degradation of apoptotic cell-derived DNA in these cells are also needed for both macrophage proliferation in response to efferocytosis and anti-inflammatory macrophage reprogramming (86). This cooperation allows efferocytic macrophages to maintain a prolonged extracellular signal-regulated kinase 1/2 (ERK1/2) activation (86), necessary for macrophage proliferation (86), PGE-2 and TGF-β production (12), as well as an accumulation of LOX-5 in the cytosol (64). As a reminder, LOX-5 may act with ALOX15 to metabolize AA into LxA4. Altogether, multiple signals delivered by apoptotic cells themselves or their byproducts are involved in the reprogramming of macrophages into pro-resolving macrophages.

#### 2.4.1 Cooperation of apoptotic cells and efferocytic macrophages

As discussed, apoptotic cells and macrophages may cooperate before the internalization of apoptotic cells to generate adenosine (**Figure 2B**). In this case, adenosine results from AMP released by apoptotic cells that is metabolized into adenosine by efferocytic macrophages (44). Thus, apoptotic cells and efferocytic macrophages act in a synergistic manner to produce adenosine, a well-known mediator of resolution (5, 87). Another situation of cooperation between macrophages and apoptotic cells leads to the production of SPM (**Figure 2B**) by a mechanism called trans-cellular biosynthesis (88). Indeed, the interactions of apoptotic neutrophils or apoptotic neutrophil-derived extracellular vesicles with macrophages increase the synthesis of different SPM, such as RvD1, RvD2, and RvE2 as well as maresin-1 and protectin-D1 (89, 90). In fact, apoptotic neutrophils or their extracellular vesicles provide an intermediate precursor −derived from the precursors (e.g., eicosapentaenoic acid [EPA] or docosahexaenoic acid [DHA])− that is converted into SPM by macrophages (90). These intermediate precursors are 15- and 12-hydroxy-EPA (HEPA) derived from EPA for E-series resolvins and 17- and 14-hydroxy-DHA (HDHA) derived from DHA for D-series resolvins, protectin-1 and maresin-1 (6, 7, 88). In addition, apoptotic neutrophil-derived extracellular vesicles may stimulate macrophage SPM biosynthesis *via* a specific signaling pathway involving G-protein coupled receptor(s)(GPCR) (90). These SPM promote macrophage pro-resolving functions, including increased efferocytosis, and TGF-β and IL-10 production *via* specific GPCR (please refer to (88).

#### 2.4.2 Cooperation of apoptotic cell-derived microenvironment with efferocytic macrophages

Solute carrier (SLC) transporters have been shown to regulate efferocytosis by macrophages (91, 92) and by type 1 conventional dendritic cells (cDC1) (93). These SLC transporters participate in the exchange of a huge number of substrates (*i.e*., ions, sugars, nucleotides, amino acids) across membranes, including plasma membranes, but also intracellular organelle membranes. They control extracellular and cytosolic concentrations of substrates that modulate cellular metabolism and signaling. The expression of 33 SLC was found to be modulated after efferocytosis as analyzed by RNA sequencing (91). Four SLC have been specifically studied in the regulation of macrophage efferocytosis (91, 92). SLC2A1 (also known as GLUT1) is a transporter facilitating glucose uptake from extracellular milieu. Glucose uptake *via* SLC2A1 regulates efferocytosis (91). SLC16A1 is a plasma membrane proton-linked monocarboxylate transporter of pyruvate and lactate. This plasma membrane transporter mediates lactate release after efferocytosis. This lactate release is critical for macrophages surrounding efferocyting cells to acquire an anti-inflammatory profile. This is attested by the upregulation of *Tgfb1* and *Il10* mRNA in “bystander” macrophages (91). SLC12A4 and SLC12A2 are involved in chloride efflux and influx, respectively. SLC12A2 dampens efferocytosis, while SLC12A4 promotes it. Most importantly, SLC12A2 regulates the anti-inflammatory response induced by efferocytosis (92). Thus, efferocytosis and its associated anti-inflammatory effect are regulated by extracellular metabolites/substrates through SLC transporters implicated in carbohydrate metabolism, intracellular pH and chloride exchange.

Polyamine import may also participate in macrophage reprogramming during efferocytosis (84). Indeed, arginine-derived polyamines, namely spermidine and spermine, increase specifically in efferocytic macrophages. This polyamine increase may not result from the retention of apoptotic cell-derived metabolites after their digestion, nor from the *de novo* biosynthesis of polyamines from arginine triggered by efferocytosis. In contrast, this accumulation of polyamines may arise from the import of polyamines present in the microenvironment. A Rac1- and actin-dependent endocytic process could be responsible for this import. The blockade of this endocytic import reduces polyamine accumulation, and prevents concomitantly macrophage reprogramming (*i.e*., the inhibition of IL-1β and IL-6) induced by efferocytosis (84). The origin of spermine and spermidine present in the microenvironment of efferocytic macrophages remains to be determined. However, spermidine has been shown to be released by apoptotic cells themselves through pannexin-1 channels (37). Overall, in addition to apoptotic cells and macrophages, the microenvironment in which these cells are present may modulate macrophage reprogramming and the subsequent release of pro-resolving factors.

One has to specify that opposite results have been reported concerning ODC activity after efferocytosis [*i.e*., reduced (84) *versus* increased activity (73)]. This may explain the need of polyamine import in the setting of reduced ODC activity (84), while increased ODC activity after efferocytosis may be able to furnish apoptotic cell-derived polyamines (73). This discrepancy could be explained by the type of macrophages used in the two studies, *i.e*., “alternatively activated” M2 macrophages (73) *versus* M1 macrophages (84). Thus, the cytokine microenvironment (type 2 IL-4/IL-13 cytokines or type 1 IFN-γ cytokine) may influence macrophage metabolism and reprogramming after efferocytosis. The engagement of the IL-4 receptor-α (IL-4Rα) by type 2 cytokines (IL-4 or IL-13) together with the recognition of apoptotic cells by MerTK and Axl has been shown not only to cooperate to induce tissue repair, but also to increase *Alox15* transcript expression by mouse pro-resolving macrophages (94). The *Alox15* gene is the murine gene coding for 12/15-Lipoxygenase (12/15-LOX) (95). Its human ortholog is 15-LOX encoded by the *ALOX15* gene (95). These enzymes −human 15-LOX and mouse 12-15/LOX− mediate the oxidation of unsaturated fatty acids. Depending on its substrate (e.g., AA, or DHA), they generate different SPM, such as D-series resolvins, protectins, or lipoxins (95). Whereas mouse and human macrophages may respond differently to IL-4 (96), an increase of *ALOX15* expression in human macrophages has been reported after efferocytosis in a type 2 cytokine microenvironment (74, 96). Thus, apoptotic cell recognition in a type 2 cytokine microenvironment promotes a pro-resolving and tissue repair profile (**Figure 2C**).

A last example of the modulation of macrophage reprogramming by the microenvironment is the activity of regulatory FoxP3^+^ CD4^+^ T cells (Treg) that promote macrophage efferocytosis *via* the type 2 cytokine IL-13 (97). Treg stimulate the IL-10 signaling cascade in macrophages (97). Thus, protagonists in addition to the two key players, apoptotic cells and macrophages, may participate in macrophage reprogramming and the secretion of pro-resolving factors (**Figure 2C**).

## 3 An example of pro-resolving factors released by macrophages after efferocytosis

Several criteria/functions have been proposed to define pro-resolving mediators. Five to eight criteria characterize these mediators, according to different authors (5, 10, 98). However, all these authors agree that not all these criteria are necessary to describe a pro-resolving mediator. The most frequent criterion is the inhibition of neutrophil trafficking, which stops to fuel the onset phase of inflammation. The other main functions of pro-resolving mediators are the following: the induction of neutrophil apoptosis, stimulation of macrophage efferocytosis (that could be assimilated as “continual efferocytosis”), macrophage reprogramming toward an anti-inflammatory or pro-resolving profile, inhibition of monocyte migration and stimulation of tissue repair (5, 10, 98). Pro-inflammatory cytokine scavenging can be considered as the last criterion (5). Additional factors released by efferocytic macrophages, such as anti-inflammatory cytokines (*i.e*., IL-10 or TGF-β) are not pro-resolving mediators *per se*, but have been frequently reported in the different studies (**Figure 1** and **Figure 2**). They exert a wider array of functions than pro-resolving mediators, and these functions are not always beneficial for our body, such as fibrosis for TGF-β (99) or excessive transient immunosuppression. Another feature shared by all the different drug candidates of resolution therapy is the need for their administration at the “right place and at the right time” (7). Moreover, some SPM are highly labile and they are quickly degraded. Under physiological conditions, pro-resolving factors are produced transiently during a given time until the end of inflammation.

In the last part of this manuscript, we will focus on a new drug candidate for resolution therapy; this consists of the secretome of macrophages having ingested and eliminated apoptotic cells (100). This drug candidate emerges directly from the critical role of pro-resolving efferocytic macrophages in the resolution of inflammation. Data obtained with other pro-resolving mediators, such as SPM could be briefly analyzed to determine the relevant experimental models and the future therapeutic indications (for details, please refer to a recent review (7)). In their Table 2 (7), the authors summarize the effects of different SPM obtained in experimental models, as well as the dose used to achieve beneficial effects. The main indications are chronic diseases resulting from non-resolving inflammation, including asthma or CIA (a mouse model for RA). The authors also mention three ongoing clinical trials; two of which use pharmaceutical derivatives of RvE1 (*i.e*., RX-10045 and RX-10001) and one study using a LxA4 analog, BLXA4-Me (7). The indications are the following: signs and symptoms of dry eye (NCT00799552, available on the clinicaltrial.gov website), gingivitis (NCT02342691), and a single and multiple ascending oral dose study in healthy volunteers (NCT00941018). In addition to this review (7), a recent study reports the use of another SPM maresin-1 in experimental autoimmune encephalomyelitis (EAE), a mouse model for MS (101). Besides these studies reporting the use of SPM in animal models of chronic diseases associated with non-resolving inflammation, SPM (7) −like other pro-resolving mediators (9)− have been tested in experimental peritonitis in order to assess their impact on neutrophils present in the peritoneal exudate and the kinetics of their elimination by macrophages. This allows researchers to evaluate different criteria of pro-resolving mediators, namely inhibition of neutrophil attraction, induction of neutrophil apoptosis and stimulation of macrophage efferocytosis. Two main peritonitis models have been used to validate the different mechanisms and signaling pathways involved in macrophage reprogramming after efferocytosis. This consists in zymosan A-induced (12, 36, 44, 64, 73, 84, 86, 97) and thioglycollate-induced (9, 84, 94) peritonitis. Indeed, after the initial infiltration of neutrophils, these cells become apoptotic and this recapitulates the different steps of efferocytosis. These models of peritonitis resolve themselves spontaneously in wild type mice. Mouse ligature-induced periodontitis is another model (102) used to identify and/or test pro-resolving mediators (9), since dysregulated inflammation is considered as a major initial pathophysiological mechanism in this disease (103). Altogether, the development of new drug candidates in the setting of resolution therapy requires the assessment of the effects of these candidates in experimental models of acute and chronic inflammation (e.g., peritonitis *versus* RA or asthma). Now, we will report our own experience with the drug candidate called SuperMApo^®^.

The SuperMApo drug candidate consists of the secretome of macrophages co-cultured with apoptotic cells at a ratio of 1:5 for 48 hours (100, 104, 105). In this setting, apoptotic cells are totally eliminated in 24 hours. SuperMApo has been generated using human and mouse cells, and tested in different xenogeneic and mouse models of acute and chronic inflammation (100, 104, 105). Mouse SuperMApo is generated using thioglycollate-elicited mouse macrophages co-cultured with mouse apoptotic thymocytes (rendered apoptotic by a 35 Gray-irradiation), whereas M2 (M-CSF-treated) monocyte-derived human macrophages cultured with human apoptotic lymphocytes allow us to generate human SuperMApo (100). Our first experiments showed that SuperMApo could be a pro-resolving complex biological drug candidate limiting inflammation in the acute inflammatory model of peritonitis (**Table 1**). The SuperMApo drug candidate demonstrates pro-resolving properties in the thioglycollate-induced peritonitis model (100). Moreover, SuperMApo enhances the efferocytic capacity of macrophages both *in vivo* and *in vitro* (100). Several groups concur that this represents one criterion for pro-resolving mediators (5, 10, 98). Furthermore, SuperMApo induces macrophage reprogramming (100), which is another criterion for pro-resolving mediators. Pro-resolving properties have also been identified in xenogeneic thioglycollate-induced peritonitis using human SuperMApo administrated in immunodeficient NOG mice reconstituted with both human peripheral blood mononuclear cells (PBMC) and polymorphonuclear neutrophils (PMN) (100). Finally, SuperMApo stimulates tissue repair in a mouse model of wound healing (104)(**Table 1**). Thus, SuperMApo exhibits at least three criteria of pro-resolving mediators and could be considered as a promising pro-resolving complex biological drug candidate.

**Table 1:** Consequences of SuperMApo treatment in different experimental models of acute and chronic inflammation.

Experimental model*	Conditions of administration (route, timing,…)	Main effects	Final results	Reference

**Models of chronic inflammatory diseases resulting from non-resolving inflammation**					
Collagen-induced arthritis (DBA/1 mice)	IV or IP – 10 injections (200 µL/mouse) each day for the first 5 injections, then, every 2 days or when SuperMApo is lyophilized and concentrated 5 times: 2 injections at two days apart – treatment starts at day 35 after CIA induction	- generation of collagen-specific Treg - pro-tolerogenic reprogramming of macrophages and pDC - mediated at least by TGF-β - no immunosuppressive effect (as attested by allogeneic skin graft rejection and survival after CLP)	- therapeutic effect on established arthritis up to 60 days following administration	(100)
DSS-induced colitis (C57Bl/6 mice)	IP – 2 injections (1 mL/mouse) the day of the first DSS cycle and 48 hr later		- improved endoscopic score and colon length - reduced clinical score	(104)
T cell transfer-induced colitis (RAG2^−/−^C57Bl/6 mice)	IP – 2 injections (1 mL/mouse) at day 10 after T cell transfer and 48 hr later	- improved intestinal barrier integrity - increased cell proliferation within the intestinal crypts - increased activation of colonic fibroblasts - decreased expression of *Fn1* mRNA coding for extracellular matrix-associated fibronectin	- improved endoscopic and histological score, as well as colon length - reduced weight loss, and clinical score	(104)
DSS-induced xenogeneic colitis (human PBMC/NSG mice)	IP - 2 injections at days 14 and 16 (1 mL/mouse, 3 times concentrated human SuperMApo)		- improved survival and endoscopic score - reduced weight loss and clinical score	(100)
Cancer-induced chronic inflammation (EL4 mouse lymphoma line/C57Bl/6 mice)	IP – 2 injections (1 mL/mouse, 2 days apart) 7 days after EL4 injection	- increased macrophage mobilization in the tumor sites - reduced circulation of myeloid-derived suppressive cells - increased IFN-γ-specific anti-tumor response	- reduced cancer progression and dissemination	(105)
**Models of acute inflammation**					
Thioglycollate-induced peritonitis (C57Bl/6J mice)	IP – 1 injection at day 0	- enhanced efferocytic capacities of macrophages - anti-inflammatory macrophage reprogramming	- improved resolution	(100)
Thioglycollate-induced peritonitis (human PMBC and PMN/ NOG mice)	IP – 1 injection at day 0 (human SuperMApo)	- enhanced efferocytic capacities of macrophages - anti-inflammatory macrophage reprogramming	- improved resolution	(100)
Xenogeneic GvHD (human PBMC/NOG mice)	IP - 1 injection (1 mL/mouse, 5 times concentrated human SuperMApo) at day 0		- improved survival - reduced clinical score	(100)
**Model of wound healing/tissue repair**					
Biopsy forceps-wounded colonic mucosa model	IP – 2 injections (1 mL/mouse) the day of injury and 48 hr later	- increased proliferating (Ki67^+^) intestinal cells - increased colonic fibroblast activation (α-SMA^+^) with reduced expression of extracellular matrix associated gene *Fn1* - Limited destruction of intestinal barrier (Reg3γ, serum FITC-dextran)	- increased wound healing (assessed by video-endoscopy)	(104)

*mouse strain is provided for each experimental model. Abbreviations: α-SMA, α-smooth muscle actin; CLP, cecal ligation and puncture-induced sepsis; DSS, dextran sodium sulfate; IP, intraperitoneal; *Fn1*, the gene coding fibronectin; FITC-dextran, fluorescein-isothiocyanate-labeled beads; GvHD, graft-*versus*-host disease; IV, intravenous; NOG mice, *NOD.Cg-Prkdc^scid^Il2rg^tm1Sug^/ShiJic* mice; NSG mice, *NOD.Cg-Prkdc^scid^Il2rg^tm1Wjl^/SzJ* mice; PBMC, peripheral blood mononuclear cells; pDC, plasmacytoid dendritic cells; PMN, polymorphonuclear neutrophils; Reg3γ, regenerating islet-derived 3γ; Treg, regulatory FoxP3^+^ CD4^+^ T cells.

Concerning experimental models of chronic diseases associated with non-resolving inflammation, SuperMApo has been tested in the mouse model of RA, CIA (100), as well as in different models of IBD, including dextran sodium sulfate (DSS)-induced and naive T cell transfer-induced colitis (104), as well as DSS-induced xenogeneic colitis (100). The capacity to reduce chronic inflammation has also been evaluated in the setting of cancer (105). SuperMApo has also been tested in xenogeneic graft-*versus*-host disease (GvHD) (100), and a biopsy forceps-wounded colonic mucosa model (104). The main results are summarized in **Table 1** together with the conditions of administration (e.g., route and timing of administration, dose, etc.). A therapeutic effect (*i.e*., the reduction of the clinical score in already established diseases) has been achieved in CIA, naive T cell-transfer-induced colitis and DSS-induced xenogeneic colitis. Thus, SuperMApo is able to control ongoing disease, clearly important for the clinical situation. Furthermore, based on data obtained in xenogeneic GvHD (**Table 1**), SuperMApo could be used to prevent acute GvHD, a major complication of allogeneic hematopoietic cell transplantation (106). Since the transplantation is always a scheduled procedure in patients, SuperMApo could be administered at the time of transplantation to reduce this GvHD and the associated morbidity and mortality (106). Altogether, this demonstrates that SuperMApo is a potential new resolution therapy for chronic diseases associated with non-resolving inflammation, as well as acute GvHD.

The SuperMApo secretome contains large quantities of the anti-inflammatory cytokines TGF-β and IL-10, the IL-1 antagonist IL-1RA, as well as three chemokines CCL5, CXCL2 and CCL22 (100). TGF-β present in SuperMApo plays a crucial role, as demonstrated by blockade and depletion experiments (100, 104). However, other factors present in SuperMApo are needed. The administration of the six factors mentioned above, as recombinant proteins, even used 3 times concentrated, does not recapitulate the therapeutic effect of SuperMApo in CIA (100). The presence of cofactors complexed with TGF-β in SuperMApo has been identified using biochips coated with anti-TGF-β monoclonal antibody, surface plasmon resonance experiments, and mass spectrometry analysis. Apoliprotein E, the complement component C1q, macrophage metalloelastase MMP12, Thbs1 and transthyretin are associated with TGF-β within SuperMApo. However, their administration together with recombinant TGF-β again has no therapeutic effect on CIA, in contrast to the administration of SuperMApo (100). In addition, TGF-β present in SuperMApo is critical to treat experimental colitis (104). Nevertheless, other growth factors present in this secretome of macrophages eliminating apoptotic cells participate in this effect. This consists of insulin-growth factor-1 (IGF-I) and vascular endothelial growth factor (VEGF) (104). Yet, TGF-β, IGF-I and VEGF participate in intestinal mucosal healing induced by SuperMApo; but they are not sufficient to resolve global intestinal inflammation and do not replace SuperMApo (104). Overall, SuperMApo contains multiple factors (approximately 500 factors), which act together to exert a therapeutic effect with TGF-β playing a central role. Some of these factors have been reported to be released by efferocytic macrophages and to exert anti-inflammatory functions (*i.e*., TGF-β (12, 19, 53, 67, 86), IL-10 (46, 53, 54, 71, 86, 97) or IL-1RA (107)). Macrophages are able to produce C1q (108), and this complement component induces the anti-inflammatory reprogramming of macrophages (63). Among the three chemokines identified in high amounts in SuperMApo, CCL5 and CXCL2 may exert pro-inflammatory functions. However, previous transcriptomic analysis of different mouse macrophage subsets shows that pro-resolving macrophages are enriched in *Ccl5* compared with pro-inflammatory macrophages (36). CCL5 may participate in tissue regeneration by recruiting stroma cells *via* CCR1 (109). Moreover, CCL5 released by M2 macrophages may improve skin wound healing (110). In contrast, the increased production of CCL5 together with type I interferon after fungal infection may be responsible for impaired mucosal healing in Crohn’s disease patients and in mice (111). CCL5/RANTES is chemotactic for type 1 (Th1) CD4^+^ T cells, monocytes, dendritic cells, and NK cells *via* the expression of its receptors CCR1 and/or CCR5 (112–115) and thus may mediate pro-inflammatory effects. In the same way, CXCL2 induces the recruitment of neutrophils (116), which may fuel the inflammatory response. Transthyretin, an amyloidogenic protein found complexed with TGF-β in SuperMApo, may also promote a pro-inflammatory response. Indeed, aggregated transthyretin stimulates the progression of osteoarthritis in mouse models. However, this requires the intra-articular injection of aggregated purified transthyretin. The same injection of non-aggregated transthyretin does not induced synovitis (117). Alternatively, the factors identified in SuperMApo may represent pro-resolving mediators, such as Thbs1 (118). Until now, efforts to use the association of multiple recombinant proteins found in SuperMApo to treat chronic inflammatory diseases have been a failure. At this stage, the complete secretome in its native form should be used to resolve uncontrolled inflammation.

Concerning the mechanisms of action, the interactions of SuperMApo with innate immune cells, adaptive immune cells and cells involved in tissue repair have been studied. This has been done *in vivo* in experimental models, but also in relevant *in vitro* assays. In peritonitis models, SuperMApo stimulates the recruitment of both human and mouse neutrophils (100). This could be related to the high amount of CXCL2 found in SuperMApo (100), since CXCL2/MIP-2 has been shown to induce mouse neutrophil recruitment in the peritoneum (116). Thus, SuperMApo exerts a pro-inflammatory function by attracting neutrophils, potentially *via* CXCL2. However, the promoting effect of SuperMApo on macrophage efferocytosis may allow these cells to rapidly eliminate apoptotic neutrophils and to accelerate the resolution phase of inflammation, as observed in the peritonitis models (100). In addition, SuperMApo administration promotes macrophage reprogramming in the CIA model (100). This means that factors released by efferocytic macrophages (*i.e*., SuperMApo) are able to confer a pro-resolving phenotype to activated pro-inflammatory macrophages. This effect is dependent on TGF-β present in SuperMApo (100). However, as mentioned above, other factors may participate in this effect. One may hypothesize that lactate released by efferocytic macrophages may be one of these factors, since this metabolite has been reported to transfer macrophage reprogramming to “bystander” non-efferocytic macrophages (91). This mechanism has been proposed to explain the reprogramming of TAM (119). SuperMApo favors plasmacytoid dendritic cell (pDC) reprogramming in the CIA model, and these cells are involved in collagen-specific Treg induction observed after the administration of SuperMApo. This effect is also dependent on TGF-β present in SuperMApo (100). This is not surprising, since we previously reported in a bone marrow transplantation model that TGF-β released by efferocytic macrophages stimulates pDC to generate Treg polarization (120). This increase of Treg observed after the administration of SuperMApo could be also due to the presence of CCL22 in this secretome. Indeed, splenic marginal zone macrophages eliminating apoptotic cells secrete high amounts of CCL22, which, in turn, induces the recruitment of CCR4^+^ Treg in the spleen (*i.e*., the site of apoptotic cell removal) (121). After the administration of SuperMApo in the CIA model, both macrophages and pDC are implicated in the generation of autoantigen (collagen)-specific Treg and this requires TGF-β (100) (**Table 1**).

The administration of SuperMApo targets also non-immune cells involved in tissue repair. This was shown in the three different models of intestinal inflammation used whatever the initial injury (mechanical [biopsy forceps], T cell-mediated or chemical [DSS]) (104) (**Table 1**). SuperMApo stimulates wound healing *via* the pro-healing properties of intestinal epithelial cells (IEC) and fibroblasts. *In vitro*, SuperMApo increases the proliferative and migratory properties of an IEC line and enhances its wound closure properties. TGF-β and IGF-I present in SuperMApo have been shown to participate in the SuperMApo-induced IEC proliferation. These two growth factors and VEGF are involved in the *in vitro* effect of SuperMApo on IEC migration (104). Moreover, SuperMApo allows IEC to acquire efferocytic properties (104). Boosting apoptotic cell clearance of colonic epithelial cells has been previously shown to dampen intestinal inflammation (122). However, it remains to be determined whether the increased efferocytic properties conferred by SuperMApo to a non-professional phagocyte, here IEC, may result from the presence in SuperMApo of opsonins (e.g., MFG-E8, GAS6 or C1q) or macrophage-derived extracellular vesicles −as previously reported for airway epithelial cells in the lungs (123).

The wound repair process occurs in three overlapping, but distinct phases. The proliferation and remodeling phases follow the inflammation one (124). TGF-β is a key activator of fibroblasts, which correspond to the central cellular effectors of fibrosis and tissue repair (99, 125). During the wound healing process, fibroblasts migrate in order to close the wound and they become activated. They differentiated into myofibroblasts with the acquisition of microfilament bundles constituted by α-smooth muscle actin (α-SMA) (126). SuperMApo stimulates the *in vitro* pro-healing properties of colonic fibroblasts, *i.e*., their migration capacity in a wound-healing scratch assay and their contractibility (in a contraction assay using collagen culture gel). Furthermore, fibroblasts exposed to SuperMApo exhibit an activated phenotype (as attested by an increased expression of α-SMA) but demonstrate reduced pro-fibrotic functions (*i.e*., a limited expression of extracellular matrix genes *Fn1*, *Col1a1*, and *Col3a1*). The *in vitro* migration properties of colonic fibroblasts in response to SuperMApo are reduced when one of the three growth factors identified in SuperMApo (*i.e*., TGF-β, IGF-I, or VEGF) is depleted. These data reporting the role of growth factors in the effect of SuperMApo fit well with previous data showing that IGF-I and TGF-β induce fibroblast proliferation, and that TGF-β promotes fibroblast migration (99). Some of the *in vitro* data have been confirmed *in vivo* using the biopsy forceps-wounded colon model. This is the case of the proliferating properties of SuperMApo on intestinal cells, fibroblast activation and the reduced expression of the *Fn1* gene (104) (**Table 1**). Overall, SuperMApo exhibits tissue repair properties by targeting IEC and fibroblasts.

Resolution therapy has been proposed to be used in cancer (127), which belongs also to chronic inflammatory diseases associated with non-resolving inflammation (1). We observed that SuperMApo reduces tumor cell dissemination to the blood and mesenteric lymph nodes (105). Moreover, SuperMApo increases specific anti-tumor T cell responses. This increase of specific anti-tumor IFN-γ responses induced by the administration of SuperMApo was found correlated to the induction of macrophages highly expressing MHC class II molecules (105). The transcriptomic analysis of different macrophage subsets identified that mouse pro-resolving macrophages are enriched for genes coding for antigen processing and presentation (MHC class II genes [*H2-Aa*]) in comparison with naive macrophages (36). Thus, the preoperative administration of SuperMApo could be tested in cancers with an important inflammatory component. However, the mechanisms of action used by SuperMApo in this setting should be further deciphered.

Data obtained in experimental models are sometimes difficult to transpose to clinical settings. One explanation could be the difference between species concerning certain immune cell subsets. This has been reported concerning efferocytic macrophages. This concerns in particular the macrophage response to IL-4 (96), arginine metabolism (85) and the LXR signaling pathways that differ between human and mouse macrophages (23). Several studies considering anti-inflammatory macrophage reprogramming and the resulting pro-resolving factors compared mouse and human macrophages (12, 84, 86). Identical mechanisms were found, except for arginase-1 (73). Similar pro-resolving mechanisms are also reported in independent studies, such as the induction of *ALOX15* or of its murine ortholog *Alox15* by the simultaneous recognition of apoptotic cells in a type 2 cytokine microenvironment (*i.e*., IL-4 or IL-13) (74, 94). We tested human SuperMApo in xenogeneic models and encouraging results were obtained (100) (**Table 1**). Based on SuperMApo content, potential adverse effects may occur after its administration. These may include excessive immune suppression or a pro-inflammatory effect related to factors present in SuperMApo. Despite the presence of high levels of TGF-β and IL-10, no immunosuppressive effect has been observed after the administration of SuperMApo in the CIA model. SuperMApo-treated mice are still able to reject a skin allograft and resist to sepsis-induced mortality in the same way as untreated mice (100). SuperMApo contains factors that may exert both pro-inflammatory and pro-resolving functions. This is the case of chemokines, CCL5 and CXCL2 or other factors such as transthyretin. To date, we did not observe any pro-inflammatory effects in the different experimental models used until now (100, 104). However, we should continue to carefully monitor for any potential pro-inflammatory consequences. An argument in favor of the initiation of clinical trials is data obtained with human fibroblasts isolated from patient colon biopsies. Inflamed lesion-derived human fibroblasts demonstrate *in vitro* proliferation and enhanced wound closure capacities in response to SuperMApo (104). Altogether, this supports the use of SuperMApo in clinical settings (e.g. RA and IBD). We propose to infuse SuperMApo intravenously. To date, the precise infusion regimen in patients remains to be determined.

## 4 Conclusion

The resolution of inflammation is currently identified as an active process resulting from the effect of pro-resolving factors. These factors, including pro-resolving mediators, can be used therapeutically (3–5, 7) to treat chronic inflammatory diseases resulting from non-resolving inflammation (1). New treatments are also required in chronic diseases for which several therapeutic options exist, such as RA. While the management of RA has dramatically changed in the last 20 years with the use of TNF inhibitors, a substantial proportion of patients treated with these anti-TNF therapies still exhibits an inadequate response and does not achieve remission. Moreover, some patients develop undesirable side effects, such as infections. Thus, new therapies are still needed in RA (2). Our encouraging results obtained with SuperMApo in CIA support its use in RA. One may also extend the indications of resolution therapy to acute inflammatory diseases with non-resolving inflammation, such as the severe form of severe acute respiratory syndrome coronavirus (SARS-CoV2) infection (128). Indeed, the blockade of anti-inflammatory macrophage reprogramming by SARS-CoV2-infected apoptotic cells has been recently reported (128). The SARS-CoV2 hyper-inflammatory syndrome (129) could result from the absence of macrophage reprogramming after efferocytosis of SARS-CoV2-infected dying cells. Severe SARS-CoV2 infections may therefore be considered as another disease resulting from non-resolving inflammation (1), and could be treated with resolution therapy (as recently proposed (130)). SuperMApo constitutes a new pro-resolving complex biological drug candidate in the therapeutic arsenal of resolution therapy.

As discussed (section 3), resolution therapy has been proposed to be used in cancer (127). Indeed, aspirin has been shown to trigger the production of different SPM (e.g. RvD1 or LxA4), which stimulate cancer resolution by targeting macrophage subsets (131). The preoperative, but not postoperative, administration of a NSAID (*i.e*., ketorolac) alone or associated with resolvins has been shown to eliminate micrometastases in different tumor-resection models (132). Furthermore, the preoperative stimulation of resolution using SPM, and more particularly D-series resolvins (RvD2, RvD3, and RvD4), inhibits metastases and promotes T cell responses (132). A recent review discusses the role of SPM to reeducate TAM in order to fight cancer (127). SuperMApo could be an additional therapy to limit cancer-induced chronic inflammation.

From a mechanistic point of view, the results currently obtained with SuperMApo in the different experimental models suggest that the effect of SuperMApo is disease-specific, targeting different pathogenic cell subsets. SuperMApo particularly affects innate and T cell responses in CIA, while intestinal-resident cells involved in tissue repair are the main target of SuperMApo in colitis (**Table 1**). This may reflect the different pathophysiological mechanisms of these two diseases and their different locations (*i.e*., the joint *versus* the colon). Concerning the pro-healing properties of SuperMApo, these different mechanisms may be linked to fibroblast heterogeneity between organs (125, 133, 134). The evaluation of the administration of SuperMApo in experimental models of chronic diseases affecting other organs, for instance, EAE, could allow us to investigate these issues. Deciphering immune mechanisms resulting from the secretome of efferocytic macrophages may shed light on the role of efferocytic macrophages, their interactions with partner cells (e.g., neutrophils, fibroblasts or Treg) in chronic inflammation. SuperMApo may also enable to identify new pro-resolving factors.

An important and necessary future step will be to improve the characterization of SuperMApo. While TGF-β seems to be the key player in both CIA and colitis, its precise mechanism remains to be determined, in particular its association with other factors that bind to several proteins (e.g., the opsonins C1q or Thbs1). TGF-β is known to participle in the resolution of inflammation by exerting immune regulatory functions, which are critical for the return to homeostasis. However, it is not clear whether it acts directly by inducing leukocyte apoptosis and by decreasing the resolution index. This cytokine plays a main homeostatic role in the control of wound healing and tissue repair (135). Macrophages are the major source of TGF-β, which is secreted in its latent form. After secretion, latent TGF-β binds to collagens or proteins with collagen-rich regions (99), such as the Complement component C1q (62). TGF-β is activated by several mechanisms and factors. Thbs1 and MMP, both present in SuperMApo, can activate latent TGF-β (99, 126). Further experiments are required to decipher the precise mechanism(s) occurring in the secretome of efferocytic macrophages (*i.e*., SuperMApo). Whereas TGF-β is profibrotic (99, 126), SuperMApo contains MMP which may participate in the destruction of extracellular matrix (124) and reduce the excessive deposition of extracellular matrix proteins. Whether SuperMApo contains cellular metabolites (e.g., lactate), SPM (or SPM intermediate precursors such as HDHA or HEPA), or extracellular vesicles remains to be determined. Lactate may participate in latent TGF-β activation (99).

To conclude, patients suffering from diseases with non-resolving inflammation have unmet medical needs. We propose that resolution therapy can help address this issue. This therapy will benefit from the significant advances performed in the understanding of efferocytosis and macrophage reprogramming. A recent research topic dealing with molecular and cellular effectors in the resolution of inflammation (136) could also facilitate the development of resolution therapy. Furthermore, the analysis of the interactions between macrophages and fibroblasts in the setting of fibrosis could also favor the development of this therapy. SuperMApo could be a way to study these interactions and to better understand the role of efferocytosis in the resolution of inflammation.

## Author contributions

All the authors performed the bibliographic search and participated in the draft of this review. PS wrote the first version of the manuscript, made the table and the figures. All authors contributed to the article and approved the submitted version.

## Funding

This work is supported by the Agence Nationale de la Recherche (ANR) under the program “Investissements d’Avenir” with reference ANR-11-LABX-0021-LipSTIC, by the Region Bourgogne Franche-Comté (support to LipSTIC LabEX), the MiMedI project funded by BPI France (grant No. DOS0060162/00), and the European Union through the European Regional Development Fund of the Region Bourgogne-Franche-Comté (grant No. FC0013440).

## Acknowledgments

We would like to thank Dr. Paul R Walker (Geneva, Switzerland) for the critical reading and editing of our manuscript, Sarah Odrion for her help in editing our manuscript, and the members of our laboratory for their work.

## Conflict of interest

FB and SP are employed by MED’INN’PHARMA, which develops a pro-resolving drug candidate called SuperMapo®. SP, FB and PS are shareholder of MED’INN’PHARMA.

The remaining authors declare that the research was conducted in the absence of any commercial or financial relationships that could be construed as a potential conflict of interest.

## Publisher’s note

All claims expressed in this article are solely those of the authors and do not necessarily represent those of their affiliated organizations, or those of the publisher, the editors and the reviewers. Any product that may be evaluated in this article, or claim that may be made by its manufacturer, is not guaranteed or endorsed by the publisher.
